# Supplementary figures and images for: Candida albicans Filamentation Does Not Require the cAMP-PKA Pathway In Vivo
Source: mBio. 2022 Apr 27;13(3):e00851-22. doi: 10.1128/mbio.00851-22 (PMC9239198; doi:10.1128/mbio.00851-22)

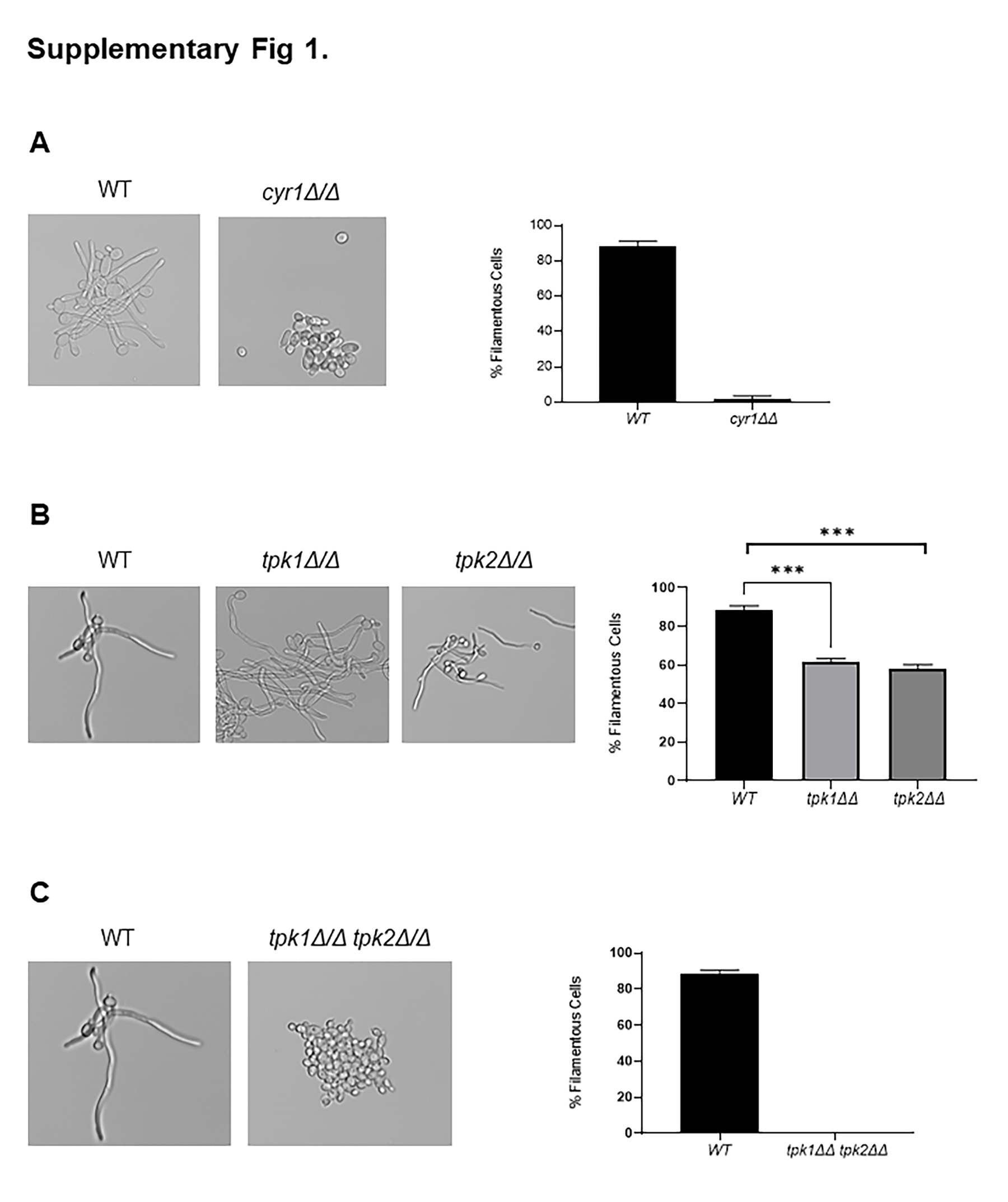

Supplement: FIG S1 [file mbio.00851-22-s0001.tif]
