# Supplementary material for: Candida albicans Filamentation Does Not Require the cAMP-PKA Pathway In Vivo
Source: mBio. 2022 Apr 27;13(3):e00851-22. doi: 10.1128/mbio.00851-22 (PMC9239198; doi:10.1128/mbio.00851-22)
Supplement: TABLE S2 [file mbio.00851-22-s0003.pdf]

Supplementary Table S2. Oligos used to construct TPK deletion mutants

Oligo Name	Oligo Sequence
TPK2 Crispr Forward #1	TTCTGGGGCAATATAATCGGGTTTTAGAGCTAGAAATAGCAAGTTAAA
TPK2 Crispr Reverse #1	CCGATTATATTGCCCCAGAACAAATTAAAAATAGTTTACGCAAGTC
TPK2 Crispr Forward #2	TGAACCACCAATCACAGCTGGTTTTAGAGCTAGAAATAGCAAGTTAAA
TPK2 Crispr Reverse #2	CAGCTGTGATTGGTGGTTCACAAATTAAAAATAGTTTACGCAAGTC
TPK2 deletion oligo Forward	TAAAGAAACTTCACATCACCAAGCTGCATCAACTGAATCAATCCAATTCGGACAGTAATTCCTTAAACTCAAACACATCAAATGATACGGCGACCACCGA
TPK2 deletion oligo Reverse	TTGACCACCTATTTTTCTTACAGTTACTATCGTTATTATTTAGTCATTTATTCATTTATGAAAGTTCATCTCCTCTCAATCGACTCCTGCATTAGGAAGC
TPK1 Crispr Forward #1	AGAAGATCCAATATATCTGGGTTTTAGAGCTAGAAATAGCAAGTTAAA
TPK2 Crispr Reverse #1	CCAGATATATTGGATCTTCTCAAATTAAAAATAGTTTACGCAAGTC
TPK1 Crispr Forward #2	AGATTAGGTAATTTACAAGGGTTTTAGAGCTAGAAATAGCAAGTTAAA
TPK1 Crispr Reverse #2	CCTTGTAAATTACCTAATCTCAAATTAAAAATAGTTTACGCAAGTC
TPK1 deletion oligo Forward	TATCCTCCTCCTTCTCCTTTCAACTTTTGAAAAAGGTGATATTATTCAACACTGTTTTTGTTTTATCAACCAAAACCAGGAATGATACGGCGACCACCGA
TPK1 deletion oligo Reverse	TGCAAATTAATTATACATCTATAAAACTAGTTATCATAATTAACATTGTTGTGCCAATAAATACAATTTTATTTTTACATCGACTCCTGCATTAGGAAGC
ARG4 Crispr Forward	atcagaactgcagatttaagGTTTTAGAGCTAGAAATAGCAAGTTAAA
ARG4 Crispr Reverse	cttaaatctgcagttctgatCAAATTAAAAATAGTTTACGCAAGTC
ARG4 Forward	ACTAACTTTGGGTCGTGTAC
ARG4 Reverse	TAGACGTGTTGTGTGTTGTG
